# Factors affecting CRISPR-Cas defense against antibiotic resistance plasmids harboured by Enterococcus faecalis laboratory model strains and clinical isolates

**DOI:** 10.1099/mic.0.001601

**Published:** 2025-09-23

**Authors:** Tahira Amdid Ratna, Belle Marco Sharon, Cesar Alejandro Barros Velin, Kelli Palmer

**Affiliations:** 1 Department of Biological Sciences, The University of Texas at Dallas, Richardson, 75080, Texas, USA

**Keywords:** antibiotic resistance, CRISPR-Cas, *Enterococcus faecalis*, horizontal gene transfer, mobile genetic element, plasmid

## Abstract

*Enterococcus faecalis* is a Gram-positive bacterium and opportunistic pathogen that acquires resistance to a wide range of antibiotics by horizontal gene transfer (HGT). The rapid increase of multidrug-resistant (MDR) bacteria including MDR *E. faecalis* necessitates the development of alternative therapies and a deeper understanding of the factors that impact HGT. CRISPR-Cas systems provide sequence-specific defense against HGT. From previous studies, we know that *E. faecalis* CRISPR-Cas provides sequence-specific anti-plasmid defense during agar plate biofilm mating and in the murine intestine. Those studies were mainly conducted using laboratory model strains with a single, CRISPR-targeted plasmid in the donor. MDR *E. faecalis* typically possess multiple plasmids that are diverse in sequence and may interact with each other to impact plasmid transfer and CRISPR-Cas efficacy. Here, we altered multiple parameters of our standard *in vitro* conjugation assays to assess CRISPR-Cas efficacy, including the number and genotype of plasmids in the donor, and laboratory model strains as donor versus recent human isolates as donor during conjugation. We found that the plasmids pTEF2 and pCF10, which are not targeted by CRISPR-Cas in our recipient, enhance the conjugative transfer of the CRISPR-targeted plasmid pTEF1 into both WT and CRISPR-Cas-deficient (via deletion of *cas9*) recipient cells. However, the effect of pTEF2 on pTEF1 transfer is much more pronounced, with a striking 6-log increase in pTEF1 conjugation frequency when pTEF2 is also present in the donor and recipients are deficient for CRISPR-Cas (compared with 4-log for pCF10). Overall, this study provides insight about the interplay between plasmids and CRISPR-Cas defence, opening avenues for developing novel therapeutic strategies to curb HGT among bacterial pathogens and highlighting pTEF2 as a plasmid for additional mechanistic study.

## Importance

The emergence of multidrug-resistant (MDR) bacteria, including MDR *E. faecalis,* limits treatment options and necessitates the development of alternative therapeutics. In these circumstances, bacterial CRISPR-Cas systems are being explored by the field to develop CRISPR-based antimicrobials. However, in many cases, CRISPR-Cas efficacy has only been assessed using laboratory model strains. More studies are required that investigate clinical isolates, as those are the intended targets for CRISPR antimicrobials. Here, we demonstrated how the number of plasmids harboured by an * E. faecalis* donor strain can affect the apparent efficacy of CRISPR-Cas anti-plasmid defence in a recipient strain. Overall, our research is important to develop improved CRISPR-based antimicrobials to combat the spread and accumulation of antibiotic resistance determinants.

## Data Availability

Supplementary material is available with the online version of this article, available through Figshare at https://doi.org/10.6084/m9.figshare.29441879 [1].

## Introduction

*Enterococcus faecalis* is a Gram-positive opportunistic pathogen [2,3]. Despite being a natural inhabitant of the mammalian gastrointestinal tract, due to faecal contamination, this pathogen is frequently found in soil, sewage, water and food [4,5]. *E. faecalis* is also a leading cause of hospital-acquired infections in the USA, especially in immunocompromised patients [6]. *E. faecalis* is considered a serious threat by the US Centers for Disease Control and Prevention due to high occurrence of resistance to a variety of antibiotics including vancomycin, a last-resort antibiotic, leaving few treatment options [7,9].

Horizontal gene transfer (HGT) disseminates antibiotic resistance genes among bacterial pathogens, including *E. faecalis* [10]. Many studies have identified mobile genetic elements (MGEs) such as pheromone-responsive plasmids (PRPs), mobilizable plasmids and transposons as means of HGT in *E. faecalis* [10,12]. Conjugation is the most studied form of HGT in * E. faecalis* [13,16]. PRPs are conjugative plasmids that can achieve very high conjugation frequencies due to their transfer mechanism, which capitalizes on sex pheromone production by recipient cells to facilitate cell contact with donors [17,19]. Thus, *E. faecalis* donor strains harbouring a PRP can transfer the plasmid to a recipient cell via conjugation and produce a transconjugant [17,20]. PRPs were first identified in *E. faecalis* and appear to exclusively replicate within the enterococci [21,22]. Examples of well-studied model PRPs are pAD1 and pCF10 [21,23, 24].

Clustered regularly interspaced short palindromic repeat and CRISPR-associated proteins (CRISPR-Cas) systems can provide sequence-specific genome defence against HGT [25,27]. This system preserves a genetic memory of past encounters with MGEs via short sequences called spacers. Upon spacer-dependent recognition of a targeted MGE, Cas-encoded nucleases can cleave and deactivate it [27,29]. In *E. faecalis*, predominantly type IIA CRISPR-Cas systems are found and characterized by the presence of type II-specific nuclease Cas9 (formerly known as Csn1) [30,32]. Genome analysis has also identified the presence of a type IIC CRISPR-Cas system in *E. faecalis* recently [33]. Thus far, four different CRISPR-Cas loci, namely, CRISPR1-Cas, CRISPR2, CRISPR3-Cas and CRISPR4, have been identified in *E. faecalis* [30,31, 34]. The plasmid recipient strain used in this study, *E. faecalis* urinary tract isolate T11RF [35,36], possesses a native CRISPR3-Cas system [31]. The T11RF CRISPR3-Cas spacer 6 has sequence identity to the model PRP pAD1 and reduces the acquisition frequency of this plasmid in T11RF [31,35].

From bioinformatic studies, we know that many multidrug-resistant (MDR) *E. faecalis* are ‘immunocompromised’ and lack functional CRISPR-Cas systems, which likely allows for the accumulation of antibiotic resistance-encoding MGEs in these strains [31,37, 38]. Using the non-MDR *E. faecalis* T11RF as a model recipient, previous works demonstrated that *E. faecalis* CRISPR-Cas confers defence against pAD1 derivatives both *in vitro* and *in vivo* (in the murine intestine) [35,39]. These experimental studies established conclusively that CRISPR-Cas can serve as an anti-plasmid defence system in *E. faecalis*. Yet, a limitation of these studies is that they assessed CRISPR-Cas efficacy against model antibiotic resistance plasmids and used laboratory model donor strains derived from the natively plasmid-free 1975 oral isolate OG1 [40]. *E. faecalis* MDR clinical isolates can possess up to six plasmids [3,41, 42], most of which have not been investigated beyond sequencing. These *E. faecalis* clinical isolates are plasmid reservoirs and donors from which antibiotic resistance spreads. Theoretically, plasmids in these donor cells may collaborate to enhance their transfer rates and/or overcome anti-plasmid defence in the recipient cell. Conjugation efficiency is influenced by a complex interplay of intracellular and intercellular interactions between distinct plasmids [43]. Indeed, it has also been reported that anti-CRISPR phages can cooperate to defeat CRISPR-Cas defense in host bacteria [44]. Hence, the number and genotype of plasmids harboured by an *E. faecalis* donor are factors that need to be explored to determine their impact on CRISPR-Cas anti-plasmid defense. Other recent research has also demonstrated that the effectiveness of a CRISPR-based antimicrobial against *E. faecalis* faecal surveillance isolates can be affected by competitive factors (such as bacteriocins) produced by the isolates [45]. This emphasizes the need to incorporate clinical isolates in research to understand CRISPR-Cas efficacy against them.

Overall, in this study, we incorporated both laboratory model strains and clinical isolates to examine the correlation between the number of plasmids present in a donor cell and the ability of CRISPR-Cas to enact its defense mechanism in the recipient. We found that the presence of multiple plasmids in a donor strain and the lack of an active CRISPR-Cas system in a recipient strain can additively confer strikingly high conjugative transfer frequencies of antibiotic resistance plasmids. Our results can be leveraged to develop enhanced CRISPR-based antimicrobials. Our research also underscores the importance of investigating additional potential factors that may influence the effectiveness of CRISPR-Cas anti-plasmid defense.

## Methods

### Bacterial strains and reagents used

Strains and plasmids used in this study are shown in Table S1 (available in the online Supplementary Material). * E. faecalis* strains were cultured in brain heart infusion (BHI) broth or agar at 37 °C without shaking, unless otherwise stated. Antibiotics were added at the following concentrations: rifampin (R), 50 μg ml^−1^; fusidic acid (F), 25 µg ml^−1^; streptomycin (S), 500 µg ml^−1^; tetracycline (Tet), 10 µg ml^−1^; spectinomycin (Sp), 500 µg ml^−1^; erythromycin (Erm), 50 µg ml^−1^; vancomycin (Van), 10 µg ml^−1^; and gentamicin (Gent), 10 µg ml^−1^. Routine PCR analysis was performed using *Taq* polymerase (New England Biolabs). Primers (Sigma-Aldrich) used in this study are shown in Table S2.

### Conjugation assays

Conjugation assays were conducted as previously described [35]. C.f.u. per millilitre was determined using the following formula: c.f.u. per millilitre=number of colony/(amount plated (ml)×dilution factor). Conjugation frequency was calculated by dividing the c.f.u. per millilitre of the transconjugants by the c.f.u. per millilitre of the donors. Raw c.f.u. per millilitre data for conjugation experiments are shown in Dataset S3. Graphs were prepared, and statistical analysis was performed using GraphPad Prism (v9.0.0).

### Selection of faecal surveillance isolates and urine isolates

Ten faecal surveillance isolates [45] and 25 urine isolates [42] were analysed for this study. First, a database was created in Geneious with the previously reported genome sequences of the 10 faecal surveillance isolates or 25 urine isolates. All CRISPR3 spacer sequences from T11RF were queried against the database using blastn. Six of the 10 faecal surveillance isolates and 17 of 25 urine isolates were identified with T11RF CRISPR3-Cas targets in either plasmids or in the chromosome with 100% nt sequence identity (Dataset S2). The presence of the expected protospacer adjacent motif sequence [35] adjacent to the target sequence was also confirmed.

### Generation of 43-2 (54 kb, pTEF2-Sp) and 101-1 (65 kb, pTEF2-Sp) donor strains and conjugation assays

*E. faecalis* V19 (pTEF2-Sp) [46] was used as donor, and faecal isolates 43-2 (**54** **kb**) and 101-1 (**65** **kb**) were used as recipients in conjugation assays to generate 43-2 (**54** **kb**, pTEF2-Sp) and 101-1 (**65** **kb**, pTEF2-Sp) donor strains. PCR was performed to confirm the plasmids present in the transconjugant. Once confirmed, 43-2 (**54** **kb**, pTEF2-Sp) and 101-1 (**65** **kb**, pTEF2-Sp) were used as donors in conjugation assays using T11RF, T11RFΔ*cas9* and T11RFΔ*cas9*+CR3 as recipients. Conjugation assays were done in biological triplicate using the previously described method [35]. Antibiotic selection plates were prepared according to the resistance gene present in the donor and recipients (Table S1). All the c.f.u. per millilitre calculation and data analysis were done as described above.

### Plasmid alignment and analysis

Plasmid tblastx alignments were performed using EasyFig 3.0.0 at default parameters. Antimicrobial resistance genes were identified using ABRicate v1.0.1, querying the ResFinder database at default parameters. pTEF2-specific genes (in comparison with pCF10) were analysed using the NCBI Conserved Domains [47], PSORTb 3.0.3 [48] and InterPro [49]. Plasmid replicon types were predicted using PlasmidFinder v2.1 at default parameters. Where plasmid replicon type was not identified using PlasmidFinder, plasmids were analysed using the NCBI Conserved Domains to identify the *rep* gene. Further typing was completed using blastn and PlasmidFinder queries at 70% identity threshold [42].

## Results

### CRISPR-Cas defence in the recipient is less effective when pTEF1 and pTEF2 are present in the donor strain, compared with pTEF1 alone

**Fig. 1.**
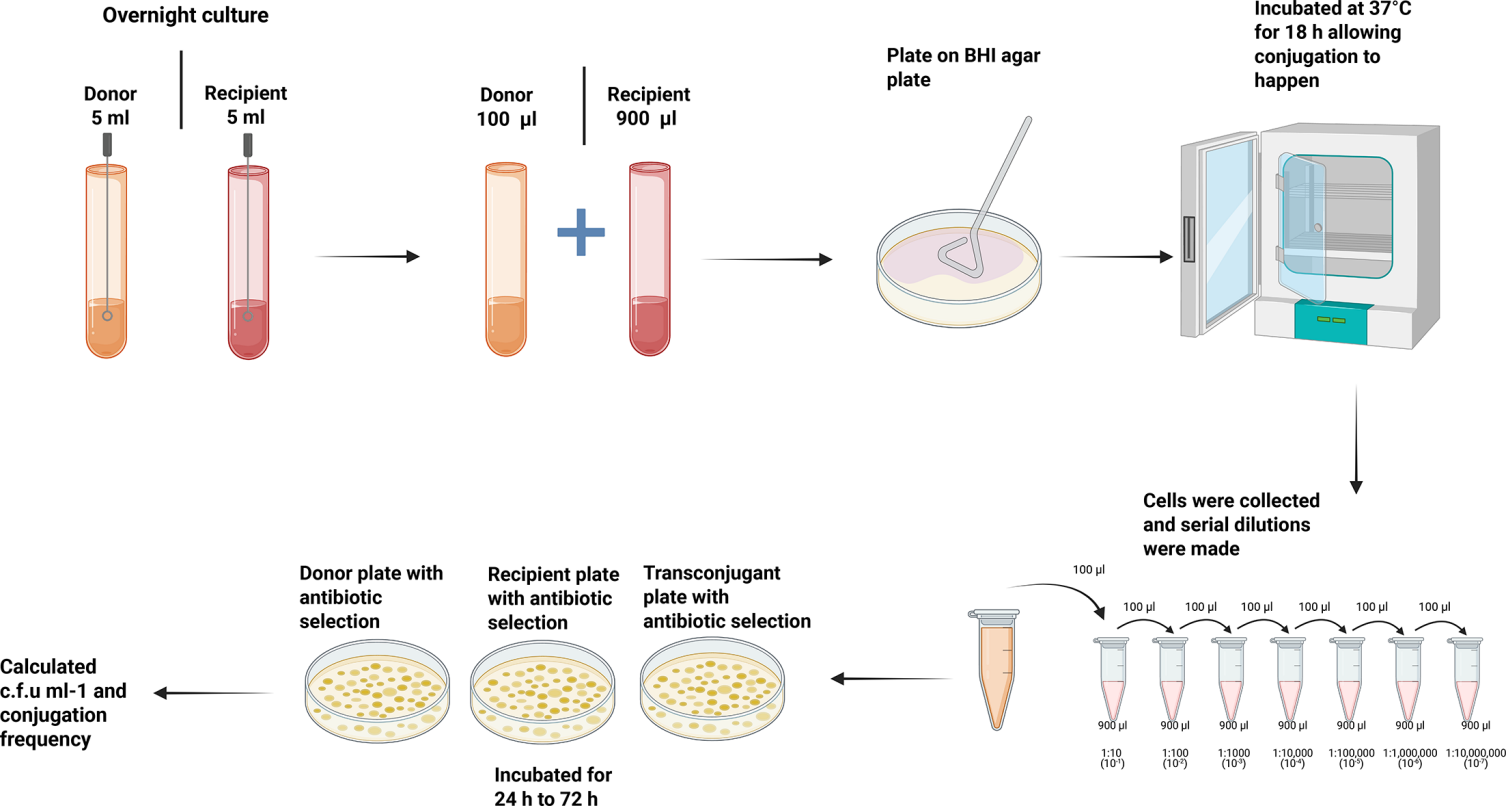
Overview of conjugation assays. On day 1, donor and recipients were inoculated in 5 ml BHI without antibiotic selection and incubated overnight at 37 °C. The next day, the cultures were diluted 1 : 10 into fresh BHI and incubated for 1.5 h at 37 °C or until log phase was reached. A 100 µl volume of donor culture was mixed with 900 µl volume of recipient culture, and the mixture was pelleted at 13,000 r.p.m. for 1 min. A 900 µl supernatant was discarded, and the remaining 100 µl of supernatant was used to resuspend the pellet. This was then plated on a BHI agar plate and incubated at 37 °C for 18 h. Cells were collected from the plate with 2 ml 1X PBS supplemented with 2 mM EDTA. This was serially diluted (10^−1^ to 10^−8^). Dilutions were plated on BHI agar plates supplemented with appropriate antibiotics to quantify donors, recipients and transconjugants. C.f.u. per millilitre was calculated after 48–72-h incubation at 37 °C and used for calculating conjugation frequency [35] (created in https://BioRender.com).

We first examined the correlation between the number of plasmids present in the donor cell and the ability of CRISPR-Cas in the recipient cell to enact its defense mechanism. We hypothesized that the presence of multiple plasmids in an *E. faecalis* donor would increase the conjugative transfer of antibiotic resistance and negatively impact CRISPR-Cas genome defense in recipients. To generate isogenic plasmid donors with varying number of plasmids, we used *E. faecalis* clinical isolate V583 as the plasmid donor and *E. faecalis* laboratory model strain OG1SSp [17,50] as the recipient strain in a conjugation assay. We chose V583 because it was among the first vancomycin-resistant clinical isolates reported in the USA [51] and contains three plasmids, pTEF1, pTEF2 and pTEF3 [3]. pTEF1 is a PRP encoding erythromycin resistance [3]. pTEF2 is also a PRP but does not encode antibiotic resistance genes [3]. pTEF1 and pTEF2 share sequence features with the model PRPs pAD1 [52] and pCF10 [19], respectively, which respond to different peptide pheromones [3]. pTEF3 is a nonconjugative broad host range plasmid [3]. Conjugation assays were conducted as described previously [35] (Fig. 1). Antibiotic-containing agar plates were used to select for OG1SSp(pTEF1) transconjugants. For all the conjugation assays, conjugation frequency was calculated by dividing the c.f.u. per millilitre of the transconjugants by the c.f.u. per millilitre of the donors. Eight transconjugants were selected for PCR to confirm their plasmid content. Two of these transconjugants had one plasmid – OG1SSp(pTEF1) – and six transconjugants had two plasmids – OG1SSp(pTEF1,pTEF2). Transfer of pTEF3 was not observed (Fig. S1). Thus, we generated our first controlled set of donors with varying number of plasmids.

**Fig. 2.**
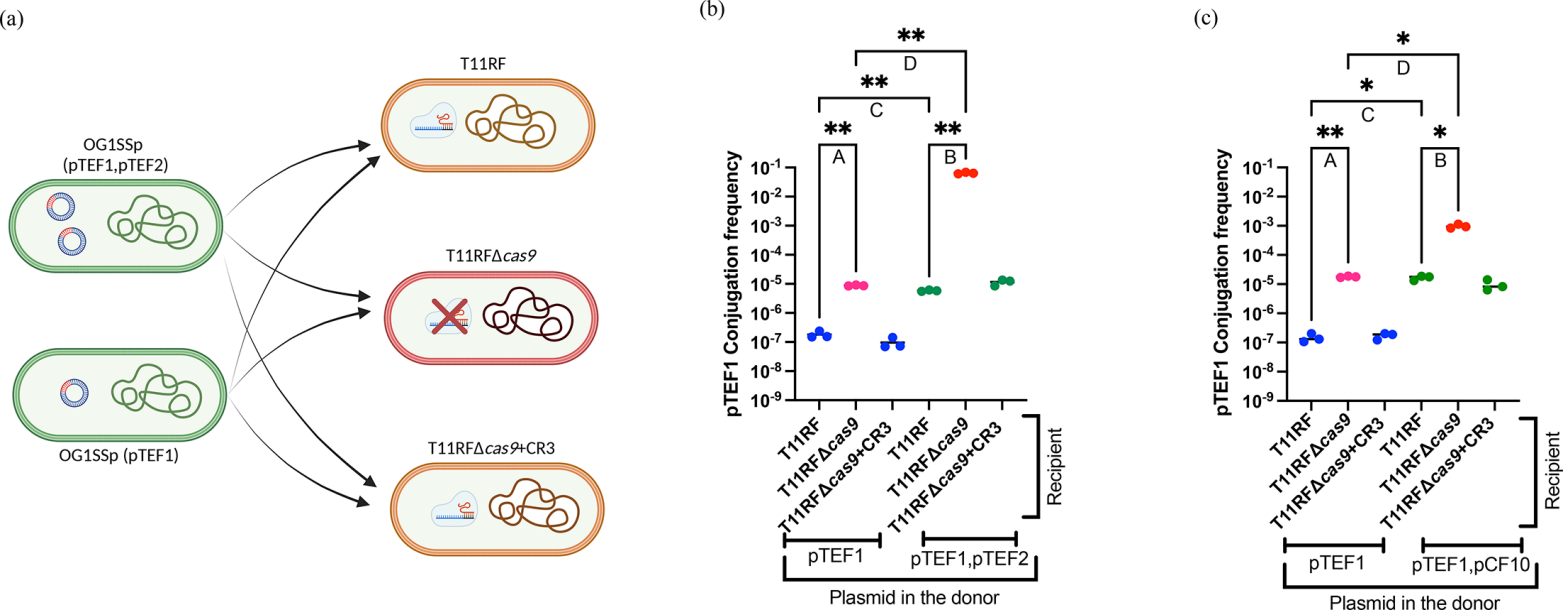
T11RF CRISPR3-Cas efficacy against pTEF1 for OG1SSp(pTEF1) and OG1SSp(pTEF1,pTEF2) or OG1SSp(pTEF1,pCF10) donor strains. (a) shows the donors OG1SSp(pTEF1,pTEF2) or OG1SSp(pTEF1) and the recipients T11RF, T11RFΔ*cas9* and T11RFΔ*cas9*+CR3 that are used in the conjugation assays for (b) (created in https://BioRender.com). In (b) and (c), conjugation frequency of pTEF1 and statistical analysis are shown. T11RF CRISPR3-Cas can provide sequence-specific genome defense against OG1SSp(pTEF1) and OG1SSp(pTEF1,pTEF2) ([b-A, b-B) [Dunnett’s T3 multiple comparisons test, **P-value=0.0029 (b-A), **P-value=0.0067 (b-B)]. However, higher conjugation frequency of pTEF1 was observed when the donor strain had both pTEF1 and pTEF2 present, compared with only pTEF1 (b-C, b-D) [Dunnett’s T3 multiple comparisons test, **P-value=0.0049 (b-C), **P-value=0.0067 (b-D)]. T11RF CRISPR3-Cas can also provide sequence-specific genome defense against OG1SSp(pTEF1) and OG1SSp(pTEF1,pCF10) (c-A, c-B) [Dunnett’s T3 multiple comparisons test, **P-value=0.0053 (c-A), *P-value=0.0360 (c-B)]. Higher conjugation frequency of pTEF1 was observed when the donor strain had both pTEF1 and pCF10 present, compared with only pTEF1 (c-C, c-D) [Dunnett’s T3 multiple comparisons test, *P-value=0.0438 (c-C), *P-value=0.0361 (c-D)].

We used the newly established OG1SSp(pTEF1) and OG1SSp(pTEF1,pTEF2) donors in further conjugation assays (Fig. 2a). *E. faecalis* urinary tract isolate T11RF [35,36] was used as the recipient strain for these experiments (Fig. 2a). T11RF is vancomycin-susceptible and often used in comparative analyses with V583 [12,31, 35]. Though T11RF and V583 share 99.5% nt identity in their core genome, T11RF lacks ~620 kb of horizontally acquired genome content present in V583 [12,31]. Spacer 6 of T11RF CRISPR3-Cas system has sequence identity with a region in pTEF1; none of the spacers have sequence identity with pTEF2, indicating that this plasmid is not targeted by the T11RF CRISPR3-Cas system. The other two recipients for this study were T11RFΔ*cas9,* where the *cas9* gene was deleted to deactivate the CRISPR-Cas system, and T11RFΔ*cas9* +CR3, where the *cas9* gene was complemented back to the Δ*cas9* mutant into a neutral site on the chromosome [35] (Fig. 2a). Conjugation assays were conducted as described previously [35] (Fig. 1). As expected, T11RF CRISPR3-Cas provided genome defense against pTEF1 for both OG1SSp(pTEF1) and OG1SSp(pTEF1,pTEF2) donors, as significantly higher pTEF1 conjugation frequency was observed in the absence of *cas9* for both (Fig. 2b-A, b-B).

We then compared the pTEF1 conjugation frequency for the two donors. We expected that, if CRISPR-Cas was equally effective against pTEF1 irrespective of pTEF2 presence, the pTEF1 conjugation frequency would be unchanged for WT T11RF recipients. However, we observed a 32-fold increase in pTEF1 conjugation frequency for WT T11RF when the donor strain had both pTEF1 and pTEF2 present, compared with only pTEF1 (Fig. 2b-C). Moreover, in the case of T11RFΔ*cas9* as the recipient strain, a striking 7,278-fold increase in pTEF1 conjugation frequency was observed when the donor strain had both pTEF1 and pTEF2 present, compared with only pTEF1 (Fig. 2b-D). We analysed the plasmid content of six T11RF and six T11RFΔ*cas9* transconjugants using PCR, finding that both pTEF1 and pTEF2 transferred to all six T11RF transconjugants and to five of six T11RFΔ*cas9* transconjugants (Fig. S2).

Our results support our hypothesis and demonstrate that CRISPR-Cas efficacy against pTEF1 is diminished when another plasmid, pTEF2, is present in the donor cell and co-transfers with pTEF1. Moreover, pTEF1 transfer frequency is extraordinarily high (a 6-log increase) when donors possess both pTEF1 and pTEF2 and recipients lack functional CRISPR-Cas defence compared with donor where only pTEF1 is present and the recipient possesses active CRISPR-Cas.

### pCF10 increased pTEF1 conjugation frequency when both plasmids were present together in a donor

pCF10 is a well-studied model PRP that shares identical conjugation genes with pTEF2 [3,19]. pCF10 encodes tetracycline resistance and is not targeted by T11RF CRISPR3-Cas [35]. Therefore, we explored whether pCF10 can ‘help’ pTEF1 in a manner similar to that observed for pTEF2. We generated another donor strain, OG1SSp(pTEF1,pCF10) and then used OG1SSp(pTEF1) and OG1SSp(pTEF1,pCF10) donor strains in conjugation assays. As expected, T11RF CRISPR3-Cas provided genome defence against pTEF1 from both donors, as higher pTEF1 conjugation frequencies were observed in the absence of *cas9* (Fig. 2c-A, c-B).

We then compared the pTEF1 conjugation frequency for the two donors. A 114-fold increase in pTEF1 conjugation frequency was observed when the donor strain had both pTEF1 and pCF10 present, compared with only pTEF1 (Fig. 2c-C). In the case of T11RFΔ*cas9* as the recipient strain, a 54-fold increase in pTEF1 conjugation frequency was observed when the donor strain had both pTEF1 and pCF10 present, compared with only pTEF1 (Fig. 2c-D). During the conjugation assay, we only tracked pTEF1 using erythromycin-containing agar. Hence, we screened transconjugants for the presence of both pTEF1 and pCF10 plasmids using erythromycin- and tetracycline-containing agars, respectively. For all transconjugants screened, pTEF1 and pCF10 plasmids were transferred together (Fig. S3). Overall, from these observations, we can say that pCF10 also increased pTEF1 conjugation frequency when present together in a donor.

We noted that Fig. 2b (for the effect of pTEF2 in donors) and Fig. 2c (for the effect of pCF10 in donors) are nearly superimposable, with the exception of the results for T11RFΔ*cas9* recipients. The presence of pTEF2 in donors conferred an additional 2-log increase in pTEF1 conjugation frequency into T11RFΔ*cas9* recipients, compared with the presence of pCF10 in donors (Fig. 2b, c). pTEF2 possesses 21 genes (in 2 clusters) that pCF10 lacks (Dataset S1). Conserved domain analysis identified putative functions including plasmid partitioning, pheromone response and modification-dependent DNA nicking for 15 of the genes; no conserved domains were identified for 6 of the genes. Further experimentation will be required to establish the basis for the extraordinarily high pTEF2-assisted pTEF1 transfer rate for T11RFΔ*cas9* recipients.

### Deletion of the aggregation substance gene *prgB* from pCF10 decreases pTEF1 conjugation frequency from a multi-plasmid donor

**Fig. 3.**
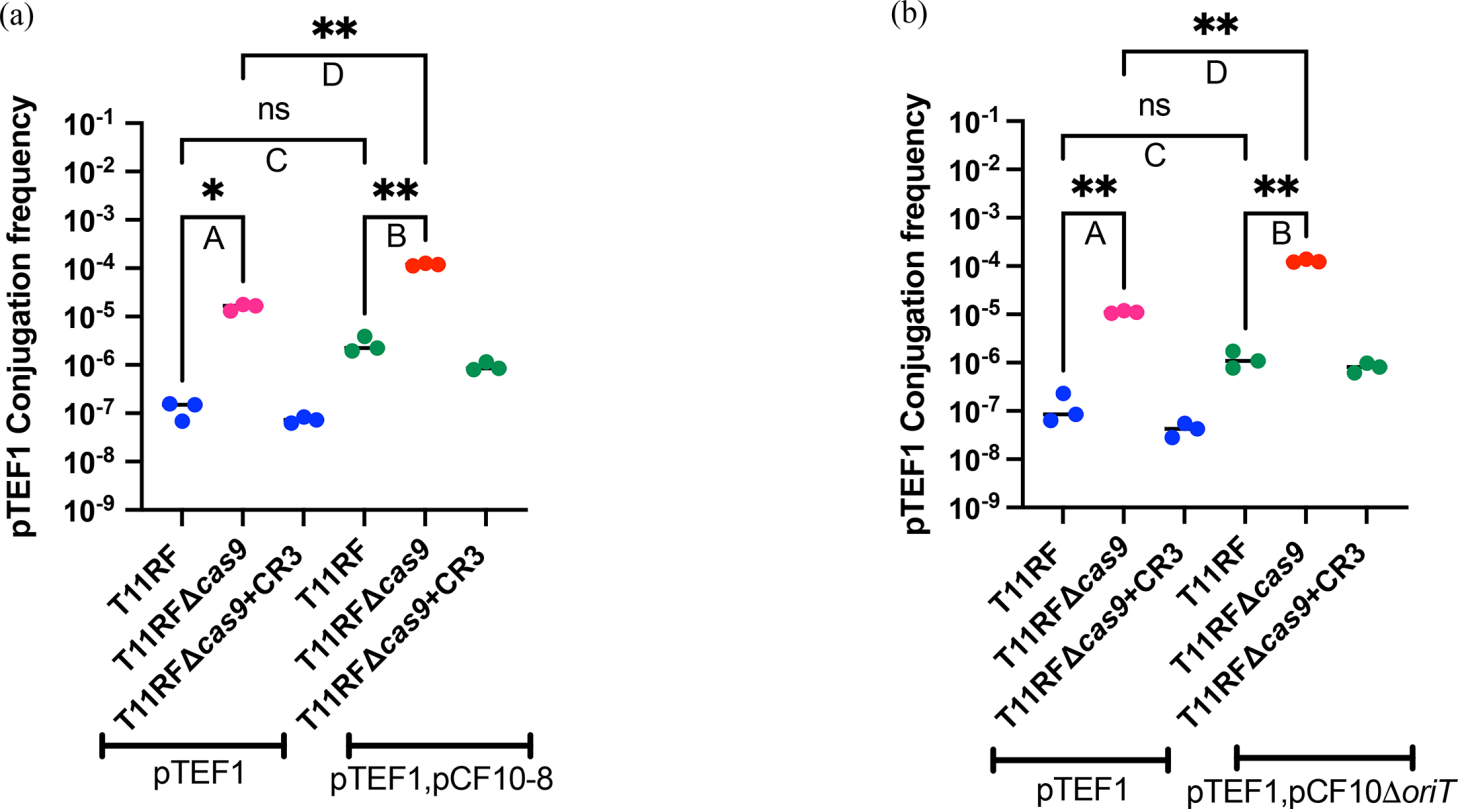
T11RF CRISPR3-Cas efficacy against pTEF1 plasmid in OG1SSp(pTEF1) and OG1SSp(pTEF1,pCF10-8) or OG1SSp(pTEF1,pCF10Δ*oriT*) donor strains. (a) and (b) show the conjugation frequency of pTEF1 and statistical analysis. CRISPR-Cas was able to provide sequence-specific genome defense against pTEF1 in both donors as higher conjugation frequency was observed in the absence of *cas9* in recipients (a-A, a-B) [Dunnett’s T3 multiple comparisons test, *P-value=0.0358 (a-A), **P-value=0.0064 (a-B)]. Higher conjugation frequency was observed when the donor strain had both pTEF1 and pCF10-8 present, compared with only pTEF1 to T11RF (a-C) or T11RFΔ*cas9* (a-D) [Dunnett’s T3 multiple comparisons test, ns *P*-value=0.2139 (a-C), **P-value=0.0088 (a-D)]. Even with the deletion of the aggregation substance gene *prgB*, pCF10 plasmid can still increase the conjugation frequency of pTEF1. CRISPR-Cas was able to provide sequence-specific genome defense against pTEF1 in both donors as higher conjugation frequency was observed in the absence of *cas9* in recipients (b-A, b-B) [Dunnett’s T3 multiple comparisons test, **P-value=0.0063 (b-A), **P-value=0.0074 (b-B)]. Higher conjugation frequency was observed when the donor strain had both pTEF1 and pCF10Δ*oriT* present, compared with only pTEF1 to T11RF (b-C) or T11RFΔ*cas9* (b-D) [Dunnett’s T3 multiple comparisons test, ns *P*-value=0.2598 (b-C), **P-value=0.0087 (b-D)]. Even with the deletion of *oriT*, the pCF10 plasmid can still increase pTEF1 conjugation frequency.

When we observed that pCF10 was able to increase pTEF1 conjugation frequency when present together in a donor, we wanted to explore which region of pCF10 might be important for this plasmid cooperativity. The pCF10 gene *prgB* encodes the aggregation substance protein Asc10 and plays an important role during conjugative transfer of pCF10 via intracellular aggregation or clumping [17,53, 54]. From previous studies, we know that the deletion of *prgB* contributes to decreased virulence and also decreased conjugation frequency of pCF10 in *E. faecalis* [53,55, 56]. Hence, we obtained a previously reported variant of pCF10 with the aggregation substance gene *prgB* deleted, referred to as pCF10-8 [53]. We generated the donor strain OG1SSp(pTEF1,pCF10-8) and then used OG1SSp(pTEF1) and OG1SSp(pTEF1,pCF10-8) donor strains in conjugation assays. CRISPR-Cas provided genome defence against pTEF1 from both donors, as higher pTEF1 conjugation frequency was observed in the absence of *cas9* in the recipient (Fig. 3a-A, a-B). When we compared the conjugation frequency of pTEF1 from the two donors, we still observed a 21-fold increase in the conjugation frequency of pTEF1 to recipient T11RF (Fig. 3a-C) or a 7-fold increase to T11RFΔ*cas9* (Fig. 3a-D), when the donor strain had both pTEF1 and pCF10-8 present, compared with only pTEF1. This means that even with the deletion of the aggregation substance gene *prgB*, pCF10-8 can still increase pTEF1 conjugation frequency. However, for WT pCF10, we observed a higher fold increase in the conjugation frequency of pTEF1 (Fig. 2c-C, c-D) than pCF10-8. Thus, we concluded that the pCF10 aggregation substance gene *prgB* is important for the increased conjugation frequency of pTEF1 from a multi-plasmid donor, but it is not the only important region under the conditions tested.

### Deletion of *oriT* from pCF10 decreases pTEF1 conjugation frequency from a multi-plasmid donor

The origin of transfer region, or *oriT*, is the region on plasmid DNA where conjugation initiates, hence playing an important role during conjugative transfer of plasmids [57]. A previous study demonstrated that the deletion of *oriT* from pCF10 significantly decreases its conjugation frequency [58]. Therefore, we obtained an *oriT*-deleted mutant of pCF10,pCF10Δ*oriT* [58] and generated the donor strain OG1SSp(pTEF1,pCF10Δ*oriT*). We used OG1SSp(pTEF1) and OG1SSp(pTEF1,pCF10Δ*oriT*) donor strains in conjugation assays with the same recipients as before. CRISPR-Cas provided genome defense against pTEF1 from both donors, as higher pTEF1 conjugation frequency was observed in the absence of *cas9* in recipients (Fig. 3b-A, b-B). When we compared the conjugation frequency of pTEF1 in two donors, we still observed a 9-fold increase in the conjugation frequency of pTEF1 to recipient T11RF (Fig. 3b-C) or an 11-fold increase to T11RFΔ*cas9* (Fig. 3b-D), when the donor strain had both pTEF1 and pCF10Δ*oriT* present, compared with only pTEF1. These results were similar to what we observed with pCF10-8 and indicate that even with the deletion of *oriT*, pCF10Δ*oriT* can still increase pTEF1 conjugation frequency. However, for WT pCF10, we observed a higher fold increase in the conjugation frequency of pTEF1 (Fig. 2c-C, c-D) than pCF10Δ*oriT*. Thus, we concluded that the *oriT* of pCF10 is also important for increased conjugation frequency of pTEF1 from a multi-plasmid donor. The potential additive effect of *prgB/oriT* double deletion was not investigated in our study but would be informative.

### T11RF CRISPR3-Cas can target clinical isolates and provide defence against resistance plasmids harboured by those clinical isolates

We included 10 previously reported hospital faecal surveillance isolates [45] and 25 previously reported urine isolates [42] in this study. At first, we checked if spacers in the T11RF CRISPR3-Cas system could target the chromosomes or plasmids of these clinical isolates. We found that 6 of 10 faecal surveillance isolates (Dataset S2) and 17 of 25 urine isolates (Dataset S2) were targeted by the T11RF CRISPR3-Cas system as evidenced by 100% nt sequence identity between spacer and target and the presence of a suitable protospacer adjacent motif (previously defined in reference [35]). The targeted plasmids and antibiotic resistance genes encoded by those plasmids are shown in Table 1 and Dataset S2. Moving forward, we will denote plasmids targeted by the T11RF CRISPR3-Cas system in bold text (for each isolate, we denote their plasmid content by their sizes in kb after the isolate name).

**Table 1.** Faecal surveillance isolates and urine isolates for conjugation assays

Faecal surveillance isolates and urine isolates	T11RF CRISPR3-array spacer that targets the plasmid or chromosome of the isolates	Resistance gene encoded in the targeted plasmids or chromosome	Rep family of the plasmids
59 (88 kb, **43 kb**)	Spacer 6	***vanHAX*** *, ermB*	rep9c, **rep9a**
43-2 (**54** **kb**)	Spacer 6	***ermB***	**rep9a**, rep2
2-1 (**149** **kb**)	Spacer 6	***ermB***	**rep9a**
133-1 (**172 kb**, 43 kb)	Spacer 7	***ermB***	**Unknown**, unknown
101-1 (**65** **kb**)	Spacer 7	***ermB***	**rep9b**
EfsC8 (**52** **kb**)	Spacer 7	***ermB***	**rep9c**
EfsC33 (**104 kb**, 72 kb)	Spacer 6	***ermB***	**rep9a**, rep9b
EfsC61 (**57** **kb**)	Spacer 7	***ant(6)-la***	**rep2**

Five faecal surveillance isolates (the first five listed) and three urine isolates (the last three) used for conjugation assays in this study are presented in Table 1. Targeted plasmids or chromosomes are shown in bold in column 1, T11RF CRISPR3-array spacer that targets the plasmid, or the chromosomes are shown in column 2 and the resistance gene encoded in the targeted plasmids or chromosomes are shown in bold in column 3. The rep family of the plasmids are shown in column 4, and the CRISPR-targeted plasmid rep family is indicated in bold [42,45]. Isolates for which conjugation was not detected are highlighted in grey.

**Fig. 4.**
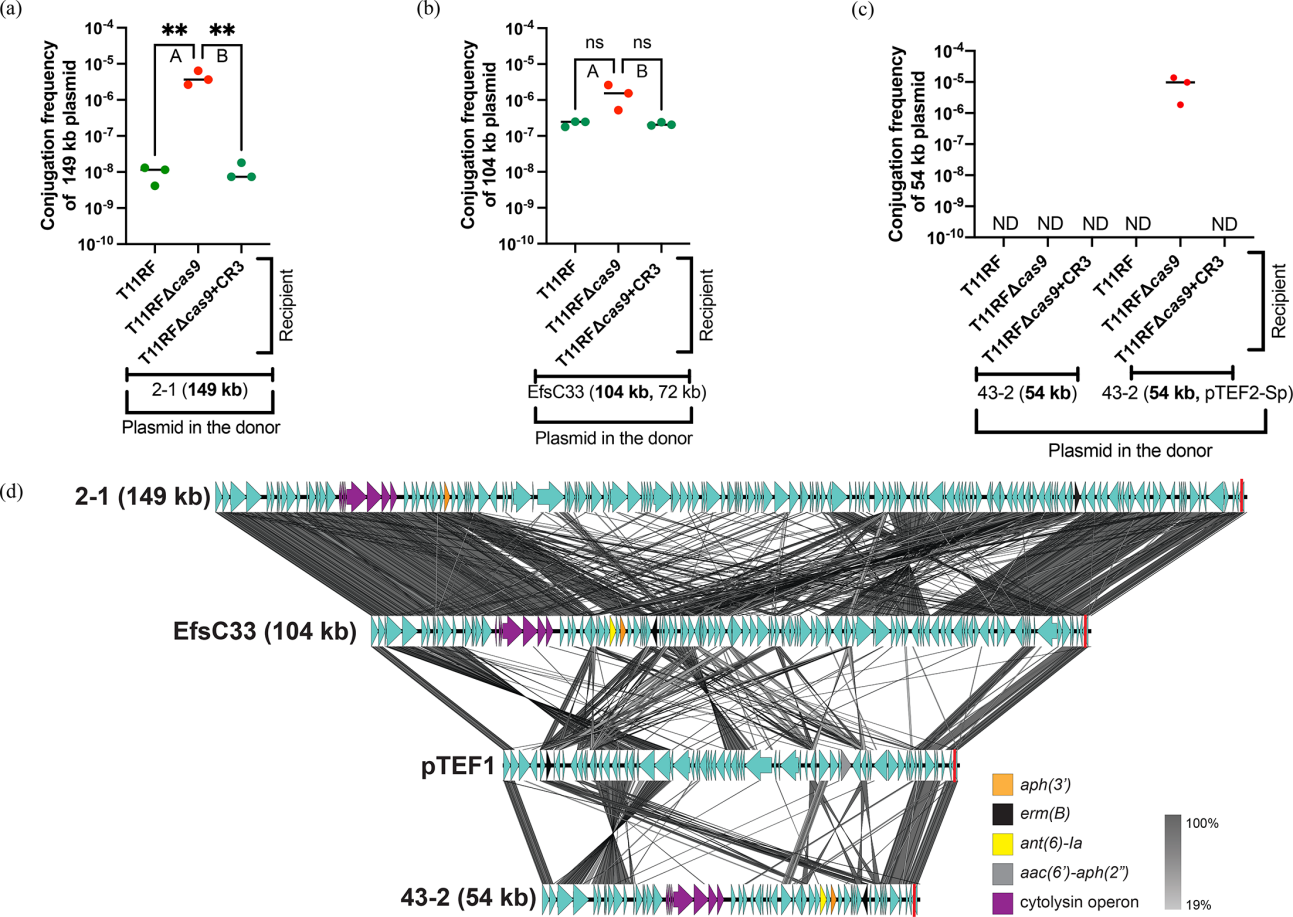
Clinical isolate plasmid donors targeted by T11RF CRISPR3-Cas spacer 6. T11RF CRISPR3-Cas decreased the rate of transfer of the **149 kb** resistance plasmid from faecal surveillance isolate 2-1 (a) [Tukey’s multiple comparison test, **P-value=0.0088 (a-A, a-B)] and the **104** **kb** plasmid from urine isolate EfsC33 (b) [Tukey’s multiple comparisons test, ns *P*-value=0.0786 (b-A), ns *P*-value=0.0765 (b-B)]. Conjugation was not detected for **54 kb** plasmid from faecal isolate 43-2 with the recipients when present alone in the donor. pTEF2-Sp mobilized the **54 kb** plasmid from faecal isolate 43-2 in the absence of *cas9* in the recipient (c). tblastx alignment of plasmids targeted by T11RF CRISPR3 spacer 6 sequence is presented in (d). The location of spacer 6 sequence is indicated with a red line. CDSs are denoted by arrows with antibiotic resistance genes and cytolysin operon genes indicated in unique colours. Lines drawn between plasmid sequences show sequence identity (per cent) (d).

**Fig. 5.**
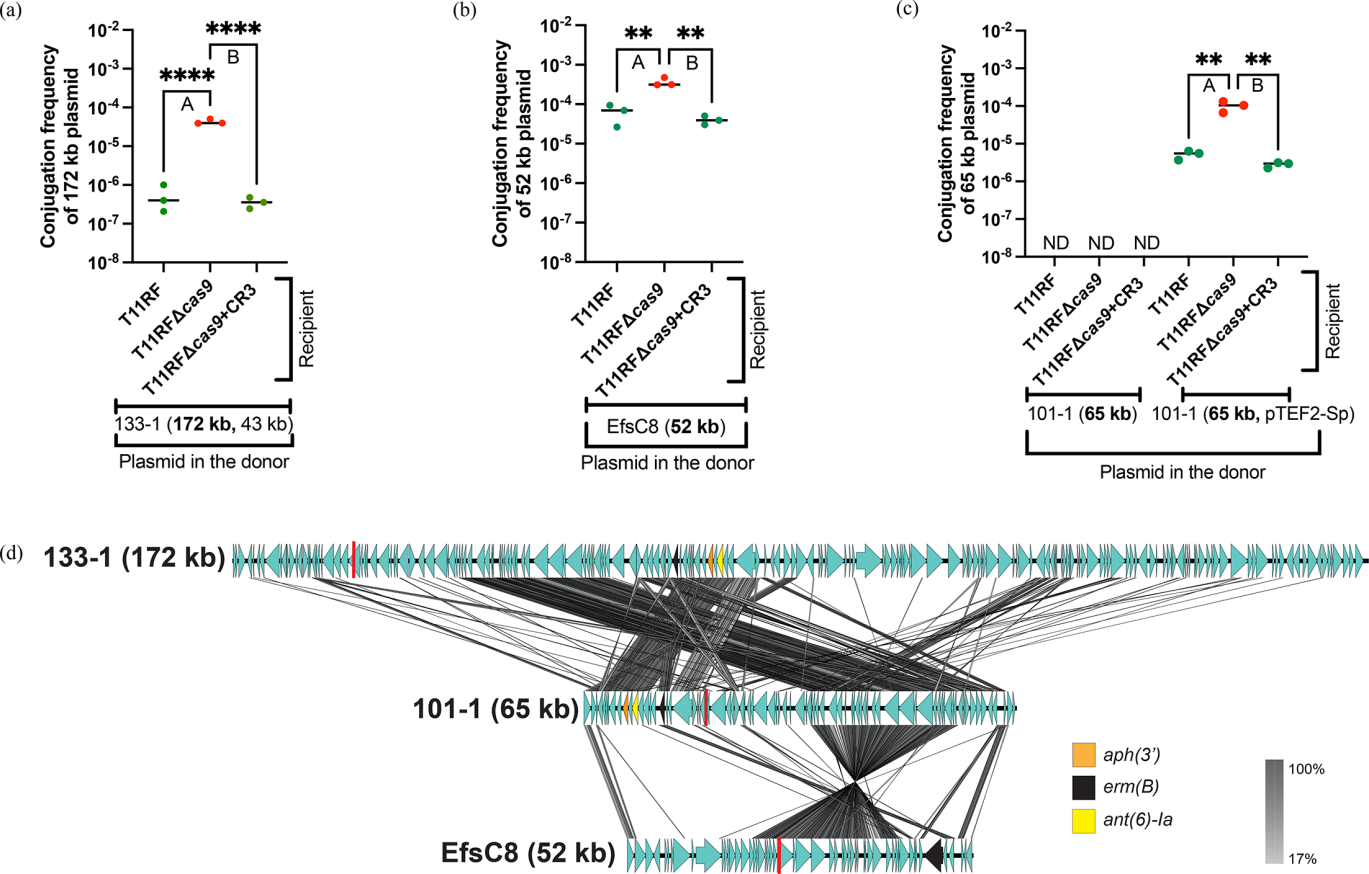
Clinical isolate plasmid donors targeted by T11RF CRISPR3-Cas spacer 7. T11RF CRISPR3-Cas was able to decrease the rate of transfer of the resistance plasmid **172 kb** from clinical faecal surveillance isolate 133-1 (a) [Tukey’s multiple comparisons test, ****P-value<0.0001 (a-A, a-B)) and **52 kb** from clinical urine isolate EfsC8 (b) [Tukey’s multiple comparisons test, **P-value=0.0016 (b-A), **P-value=0.0011 (b-B)]. Conjugation was not detected for faecal isolate 101-1 with the recipients when present alone in the donor. pTEF2-Sp plasmid mobilized the clinically resistant plasmid **65 kb** from the faecal isolate 101-1. However, T11RF CRISPR3-Cas was still able to decrease the rate of transfer of the resistance plasmid **65 kb** as higher conjugation frequency was observed in the absence of *cas9* gene (c) [Tukey’s multiple comparisons test, **P-value=0.0019 (c-A), **P-value=0.0017 (c-B)]. tblastx alignment of plasmids targeted by T11RF CRISPR3 spacer 7 sequence is presented in (d). The location of spacer 7 sequence is indicated with a red line. CDSs are denoted by arrows with antibiotic resistance genes indicated in unique colours. Lines drawn between plasmid sequences show sequence identity (per cent).

The faecal isolates 59 (88 kb, **43** **kb**), 43-2 (**54** **kb**), 2-1 (**149** **kb**), 133-1 (**172 kb**, 43 kb) and 101-1 (**65** **kb**) and urine isolates EfsC8 (**52** **kb**), EfsC33 (**104** **kb**, 72 kb) and EfsC61 (**57** **kb**) were used as plasmid donors in conjugation assays with T11RF recipients (Table 1). We chose these clinical isolates as plasmid donors because they harbour CRISPR3-targeted resistance plasmids, and thus, we were able to track those plasmids during conjugation assays. Plasmids with sequences targeted by spacer 6 and spacer 7 are shown in Figs.4  5, respectively. For faecal isolates 59 (88 kb, **43** **kb**), 43-2 (**54** **kb**) and 101-1 (**65** **kb**) and urine isolate EfsC61 (**57** **kb**), conjugation was not detected. Faecal isolates 2-1 (**149** **kb**) and 133-1 (**172 kb**, 43 kb) and urine isolates EfsC8 (**52** **kb**) and EfsC33 (**104 kb**, 73 kb) conjugated with our recipients at detectable levels. The T11RF CRISPR3-Cas system provided defense against the **149** **kb** plasmid from the faecal isolate 2-1 (Fig. 4a) with a more modest effectiveness against the **104** **kb** plasmid from the urine isolate EfsC33 (Fig. 4b). Interestingly, this was very comparable to T11RF CRISPR3-Cas spacer 7 targeted plasmids (Fig. 5). The T11RF CRISPR3-Cas system provided defense against the **172** **kb** plasmid in faecal isolate 133-1 (Fig. 5a) and a weaker protective effect against the **52** **kb** plasmid in urine isolate EfsC8 (Fig. 5b)as higher conjugation frequencies for these plasmids were observed in the absence of *cas9* in recipients.

We noted highly variable transfer rates of the CRISPR3-targeted plasmids and the magnitude of CRISPR3-Cas efficacy against them, despite the plasmids being PRPs from similar plasmid rep families. We performed plasmid alignment of the spacer 6- and spacer 7-targeted plasmids (Figs4d  5d, respectively). Our alignments corroborate conclusions reached by prior investigation of the *E. faecalis* urine isolate collection, namely, that plasmid rep typing fails to capture the genetic diversity of PRPs [42]. The variability of CRISPR3-Cas defence against these ‘wild’ plasmids is likely due to their genetic diversity and interactions with other MGEs in the donor, which together influence plasmid transfer frequency and interaction with recipient defence systems. This study marks a significant milestone as the first to demonstrate the efficacy of the T11RF CRISPR3-Cas system in targeting plasmids from recent clinical isolates.

### pTEF2 increases conjugation frequency of resistance plasmids from two faecal isolates and helps them escape T11RF CRISPR3-Cas defence

Conjugation was not detected for the faecal isolates 43-2 (**54 kb**) and 101-1 (**65 kb**) with T11RF (Figs4c  5c). We investigated what effect pTEF2 would have on the transfer of these plasmids, given our results demonstrating that pTEF2 could assist pTEF1 with transfer and apparent evasion of CRISPR-Cas defence (Fig. 2b). We made new donor strains with pTEF2-Sp (pTEF2 modified to encode spectinomycin resistance [46]). We used these new donor strains, 43-2 (**54 kb**, pTEF2-Sp) and 101-1 (**65 kb**, pTEF2-Sp) in conjugation assays with our previous recipients. We observed that pTEF2-Sp can mobilize the clinically resistant plasmids, as they conjugated to the recipients. However, T11RF CRISPR3-Cas still provided defence against the **54 kb** plasmid of the 43-2 strain (Fig. 4c) and the **65 kb** plasmid of the 101-1 strain (Fig. 5c), as in the absence of T11RF *cas9,* higher conjugation frequencies were observed. For the 43-2 donor strain, conjugation of the **54 kb** plasmid was detected only when *cas9* was deleted from the recipient strain. We did not perform the same experiment with pCF10, as pCF10 encodes tetracycline resistance, and 43-2 and 101-1 also encode tetracycline resistance in their chromosome. Therefore, we concluded that pTEF2 can increase the conjugation frequency of other resistance plasmids and help them in escaping T11RF CRISPR3-Cas defence. Our findings underscore the importance of considering plasmid cooperativity when refining CRISPR-based antimicrobial designs. Our work suggests that targeted depletion of pTEF2 from *E. faecalis* populations may specifically be useful for mitigating antibiotic resistance plasmid spread.

## Discussion

In this era of antibiotic resistance, *E. faecalis* is considered a serious threat as it can acquire a large number of resistance genes via HGT [3,10]. Bacterial CRISPR-Cas systems may be leveraged as an alternative treatment approach, providing sequence-specific cleavage of antibiotic resistance plasmids in *E. faecalis* [35,39]. Most available studies of CRISPR-Cas efficacy have used laboratory model strains and laboratory model plasmids. However, clinical isolates can behave differently than the laboratory model strains, and CRISPR-Cas efficacy can be affected by many factors [45]. Therefore, for this study, we included both laboratory model strains and clinical isolates to study previously unexplored factors that might affect CRISPR-Cas efficacy. Our hypothesis was that the number of plasmids present in a donor strain is an important factor that might affect CRISPR-Cas efficacy. Through our experiments, we showed our hypothesis is supported and found two plasmid pairs (i) pTEF1,pTEF2 and (ii) pTEF1,pCF10 that showed plasmid cooperativity. From our experiments, we saw that pTEF2 and pCF10 were able to increase pTEF1 conjugation frequency. We identified two regions, (i) aggregation substance gene *prgB* and (ii) *oriT* of pCF10, potentially important for the plasmid cooperativity as the deletion of these regions resulted in decreased conjugation frequency of pTEF1.

We included 10 clinical faecal surveillance isolates [45] and 25 urine isolates [42] in this study. We found that 6 faecal surveillance isolates and 17 urine isolates can be targeted by the T11RF CRISPR3-Cas system. When we used these clinical isolates as plasmid donors, we found that T11RF CRISPR3-Cas was able to provide defense against the resistance plasmids harboured by those isolates. To the best of our knowledge, this is the first research studying T11RF CRISPR3-Cas efficacy against a collection of clinical isolates. Interestingly, there is a significant impact of CRISPR-Cas on targeted plasmids, but the magnitude of the impact differed for different clinical isolates. There were a few clinical isolates that did not conjugate with our recipient at detectable levels. However, when we conjugated pTEF2-Sp plasmid in those clinical isolates, pTEF2-Sp was able to mobilize two clinically resistant plasmids (i) **54 kb**, pTEF2-Sp from 43-2 and (ii) **65 kb**, pTEF2-Sp from 101-1. However, the T11RF CRISPR3-Cas system was still able to provide defense against those plasmids once they were mobilized. This finding is similar to our plasmid cooperativity results of pTEF1,pTEF2 and pTEF1,pCF10. Together, these plasmid cooperativity results indicate that the first conjugative resistance plasmid lacks something essential for robust transfer, to which they are getting ‘help’ from the second plasmid, and together, they escape T11RF CRISPR3-Cas defense by dint of higher conjugation frequency. A limitation of our experimental design is we did not pinpoint the exact mechanism of this plasmid cooperativity. It could be the helper plasmid enhancing the transfer frequency of the first plasmid, the helper plasmid inhibiting the CRISPR-Cas defense against the first plasmid or both.

Overall, we conclude that the presence of multiple interacting plasmids in donor strains and the lack of an active CRISPR-Cas system in a recipient strain act additively to confer extremely high plasmid transfer frequencies in *E. faecalis*. To date, a few multi-plasmid studies have demonstrated how ecological settings influence the maintenance of individual plasmids within a multi-plasmid community, how a conjugative helper plasmid can mobilize a non-conjugative virulence plasmid and how interactions between plasmids can affect conjugation efficiency [43,59, 60]. Hence, our study opens a new direction of plasmid cooperativity and contributes towards designing novel CRISPR antimicrobials.

Apart from the plasmid number present in donor strains, there may be other factors affecting CRISPR-Cas efficacy, such as biofilm formation capacity or internal CRISPR-Cas regulation in the recipient strains. For example, transcriptional regulation of *cas9* was reported as a major factor, which can influence CRISPR-Cas genome defense in *Streptococcus pyogenes* [61]. Hence, it is necessary to continue to study potential factors that might affect CRISPR-Cas efficacy in future studies. Overall, our research is significant in its application towards designing improved CRISPR-based antimicrobials as an alternative approach to combat HGT and antibiotic resistance. Our research also emphasizes to study factors affecting CRISPR-Cas efficacy and conducting research using clinical isolates. The more information we gather on the efficacy of CRISPR-Cas, the more effectively we can harness it as an alternative treatment approach.

## Abbreviations

BHI: brain heart infusion
HGT: horizontal gene transfer
MDR: multidrug-resistant
MGE: mobile genetic element
PRP: pheromone-responsive plasmid
